# Influence of Magnetic Nanoparticles on the Focused Ultrasound Hyperthermia

**DOI:** 10.3390/ma11091607

**Published:** 2018-09-04

**Authors:** Katarzyna Kaczmarek, Tomasz Hornowski, Bernadeta Dobosz, Arkadiusz Józefczak

**Affiliations:** 1 Institute of Acoustics, Faculty of Physics, Adam Mickiewicz University, Umultowska 85, 61–614 Poznań, Poland; katarzyna.kaczmarek@amu.edu.pl (K.K.); hornaku@amu.edu.pl (T.H.); 2 Medical Physics Division, Faculty of Physics, Adam Mickiewicz University, Umultowska 85, 61–614 Poznań, Poland; benia@amu.edu.pl

**Keywords:** focused ultrasound, therapeutic ultrasound, ultrasound hyperthermia, magnetic nanoparticles, sonosensitizers

## Abstract

Ultrasound hyperthermia is a medical treatment used to increase temperature of tissues. It can be used independently or as a supportive method for an anticancer treatment. The therapeutic efficacy of focused ultrasound hyperthermia can be improved using sonosensitizers, nanoparticles enhancing the attenuation and dissipation of acoustic energy. As sonosensitizers, we propose magnetic nanoparticles owing to their biodegradability, biocompatibility, and simple positioning in tissues using a magnetic field. Focused ultrasound hyperthermia studies were performed using tissue-mimicking phantoms. Temperature changes were measured at various ultrasound powers and distances from the center of the ultrasound focus. Specific absorption rate (SAR) values, describing the power deposition in the tissues during the hyperthermia treatment, were evaluated for the center of the focus point and for various distances from it. The results show that the addition of nanoparticles increases the SAR almost two times compared to that for the pure phantom. The highest SAR is obtained in the ultrasound focus; it decreases with the increase of the distance from the focus.

## 1. Introduction

The clinical ultrasounds are acoustic waves with frequencies in the range of 1–10 MHz. Owing to their small wavelength on the order of millimeters, as well as the possibility to focus their beam in a small area, they have been employed in various biomedical applications [1,2,3]. Most commonly, ultrasounds are used in diagnostic medical imaging, referred to as ultrasonography and ultrasound tomography [3,4,5,6,7]. They are also very important in enhanced drug uptake through skin, i.e., sonophoresis, transdermal drug delivery, and lithotripsy to destroy kidney stones [2,3]. Furthermore, they have been employed in various thermal therapies. Depending on the temperature increase induced by the ultrasound waves, a few thermal therapies can be distinguished [2,8].

Hyperthermia is a medical treatment involving an increase of the tissue temperature to 42–45 °C [4,9,10]. It is commonly used for noninvasive heating of muscles and tendons [2]. Numerous biological and clinical investigations have demonstrated that hyperthermia is a very promising anticancer medical treatment. The heat generated in the cancer tissue leads to its weakening. The weakened cells are more susceptible to other cancer treatments such as radiotherapy or chemotherapy [8]. The medical procedure in which the temperature is increased to values above 45 °C is referred to as thermal ablation. It causes irreversible changes in tissues and direct destruction of cancer cells [8,9,11].

The most important advantage of ultrasound heating, compared to other external stimuli, is that the acoustic beam can be focused and localized with a millimeter precision. Therefore, it precisely interacts with a small volume of the tissue. The energy of the focused ultrasound (FUS) can be deposited in a lesion with a diameter of 1 mm [12]. This beam focusing ability minimizes the possibility of thermal damage of tissues localized near the focal area or between the focal point and transducer [5,13,14]. FUS is widely used in medicine for therapeutic purposes [3,5,14]. Depending on the energy values, FUS can be used for ablation, cell destruction, necrosis, or cell apoptosis [3,5,14]. High-temperature FUS provides a precise targeted drug delivery and targeted drug release in a local area [2,4]. In contrast to other therapies, e.g., cryoablation, the FUS therapy is noninvasive and reaches tissues and areas localized deep within the body [14]. By appropriate choice of frequency penetration depth of ultrasound in tissue can be controlled [15]. Higher frequencies produce concentrated absorbed energy but fall off quickly with depth. Lower frequencies, conversely, are not quickly absorbed and, thus, decay slowly with depth [4]. The penetration depth of ultrasound in tissue is about 20 cm for 1 MHz [4].

Recently, a combination of therapy and diagnostics referred to as theragnostic has become very popular [6,12]. Ultrasound waves are very often used in such therapies to diagnose, introduce a targeted treatment, and simultaneously monitor the response to the therapy [6,12,16]. Moreover, in order to ensure a proper temperature increase in the target area, often high-intensity focused ultrasound (HIFU) procedures are combined with magnetic resonance imaging or ultrasound imaging [3,14,17,18].

The requirement of high acoustic intensities in the thermal therapies such as FUS and HIFU often leads to negative side effects such as skin burns, pain, or discomfort [2,17,19]. Extensive studies have been performed to design and develop novel transducer technology and array designs to achieve a rapid delivery of focused sonication [12]. Another possible strategy to achieve the required temperatures without the use of high energies is to use additional materials, known as sonosensitizers, which increase the ultrasound attenuation [20,21,22,23,24]; for example, magnetic nanoparticles suspended in a carrier liquid. Magnetic nanoparticles in the form of ferrofluid cause an additional increase in the temperature owing to the additional attenuation of the acoustic wave [17,20,25,26]. Magnetic nanoparticles are good candidates to sonosensitizer the material in ultrasound hyperthermia as they are biocompatible, nontoxic, biodegradable, and commonly used in several medical applications [27,28]. Moreover, magnetic nanoparticles can be administered intravenously or intra-arterially to the patient body. They can be directly injected into tumor area [29] or delivered to the veins and then easily and precisely captured in the tumor area by an external inhomogeneous magnetic field. It is possible to obtain good nanoparticle distribution throughout tissues and organs [30]. The nanoparticles within the tumor area locally increase the attenuation of the ultrasound wave; therefore, only this region is heated, while the surrounding healthy tissue remains unaffected [31]. Simultaneously, owing to their susceptibility to the alternating magnetic field, they become a source of heat in magnetic hyperthermia [25,26]. Thus, magnetic nanoparticles can be applied as a material base for the use of synergetic effects of combined treatments (ultrasound and magnetic hyperthermia), which often show much higher efficacy than single therapy strategies. Recently, there has been a great interest in the application of multimodal hyperthermia, but according to the authors’ knowledge, nobody has tried to combine magnetic and ultrasound hyperthermia yet. However, to confirm and explain the reasons for these synergetic effects, it is necessary to conduct more studies on the same tissue phantoms in comparable conditions with the use of ultrasound and magnetic hyperthermia.

The aim of this study is to investigate the thermal effect of FUS hyperthermia enhanced by magnetic nanoparticles as a function of the acoustic power and distance from the center of the ultrasound focal zone. The specific absorption rate (SAR), which describes the rate of energy absorption by the tissue exposed to ultrasound, was calculated and used for an additional data analysis. The SAR values were used to precisely describe and visualize the effectiveness of the hyperthermia in the focal point and surrounding area.

## 2. Materials and Methods

### 2.1. Preparation and Characterization of Nanoparticles

**Figure 1:**
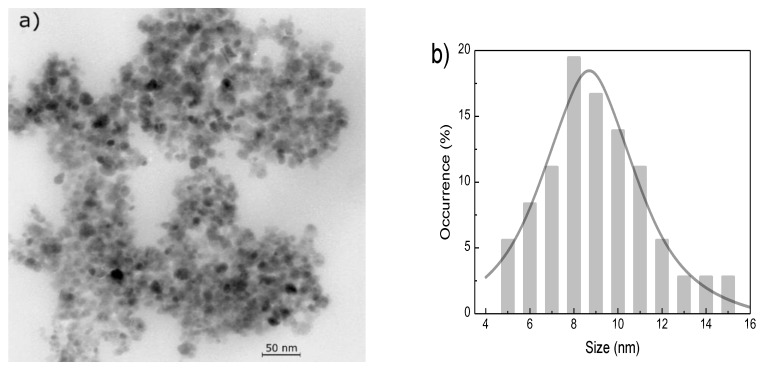
(**a**) Transmission electron microscopy image; (**b**) Magnetic-particle size distribution.

In this study, magnetic nanoparticles were used as sonosensitizers, synthetized at the Institute of Experimental Physics at the Slovak Academy of Science in Kosice, Slovakia, using an adapted procedure originally proposed by Molday [32]. The magnetic core was covered with two layers of surface active agents: sodium oleate and polyethylene glycol PEG 6000 to prevent agglomeration and ensure material biocompatibility and nontoxicity [28,29]. The concentration of the magnetic material in the prepared magnetic fluid was determined to be 23 mg/ml using ultraviolet–visible (UV–VIS) spectrophotometry (Analytik Jena AG, Jena, Germany). The average size of the magnetic core obtained from a magnetic measurement using a vibrating sample magnetometer (VSM, Cryogenic Ltd., London, UK) was 28 nm; however, the dominant part of the magnetization originated from particles with a magnetic core size of 7 nm [33]. In order to provide further insights into the characteristics of the nanomaterial, a transmission electron microscopy (TEM, JEOL Ltd., Tokyo, Japan) analysis was performed. The obtained TEM image provides supplementary information about the size and shape of individual nanoparticles. The TEM image in Figure 1 shows that the employed magnetic fluid has a polydisperse size distribution. The size of the magnetic grain estimated from the TEM measurement was 8 nm, which is in a good agreement with the nanoparticle size obtained from the VSM measurement.

### 2.2. Preparation and Characterization of Phantoms

The initial phase of many studies on ultrasound procedures on humans, including ultrasound hyperthermia, is performed on materials that mimic human tissues. Several tissue-mimicking materials such as polyvinyl alcohol, gelatin, or oil-based gel have speed of sound, attenuation, density, and acoustic impedance within the corresponding measured ranges of soft tissues [34]. In this study, we used tissue-mimicking materials made from agar powder, substance obtained from red algae. Agar forms a thermo-reversible gel in an aqueous solution; the gel remains stable over a wide temperature range. The agar-based gel has acoustic parameters (velocity of approximately 1540 m/s, density of approximately 1.0 g/cm^3^, and attenuation of approximately 0.3–0.5 dB/cm·MHz) very similar to those of the human tissue [1,35].

For the hyperthermia measurements, we prepared two types of phantoms: pure agar gel phantoms without any added scattering material and agar gel phantoms with magnetic nanoparticles. The phantoms had a cylindrical shape with a diameter of 3 cm and height of approximately 3.5 cm. The weight concentration of agar in the phantoms *ϕ_A_* was 5% (*w*/*w*), while the concentration of the magnetic nanomaterial *ϕ_M_* was 0.35% (*w*/*w*) (molar concentration: 0.0159 mol/L). The employed agar powder was characterized by the company HiMedia as a standard plate count agar (Standard Methods Agar M091-500G, Mumbai, India).

### 2.3. Electron Spin Resonance Studies

For a better understanding of the characteristics of the prepared nanomaterial and ultrasound phantoms, electron spin resonance (ESR) studies were performed. ESR is an effective method to study the properties and quality of magnetic nanoparticles as well as the impact of various conditions on these properties [36,37,38]. The technique is based on the Zeeman phenomenon, in which a splitting of the electron energy levels occurs in the sample, caused by the interaction of an external magnetic field with magnetic moments of unpaired electrons. In order to observe the ESR signal, the resonance condition has to be fulfilled:

(1) $$h\nu =g\mu_{B}B$$

where *g* is the spectroscopic splitting factor, characteristic for each paramagnetic center, *μ_B_* is the Bohr magneton, *B* is the induction of the external magnetic field, and *ν* is the microwave frequency [39].

**Figure 2:**
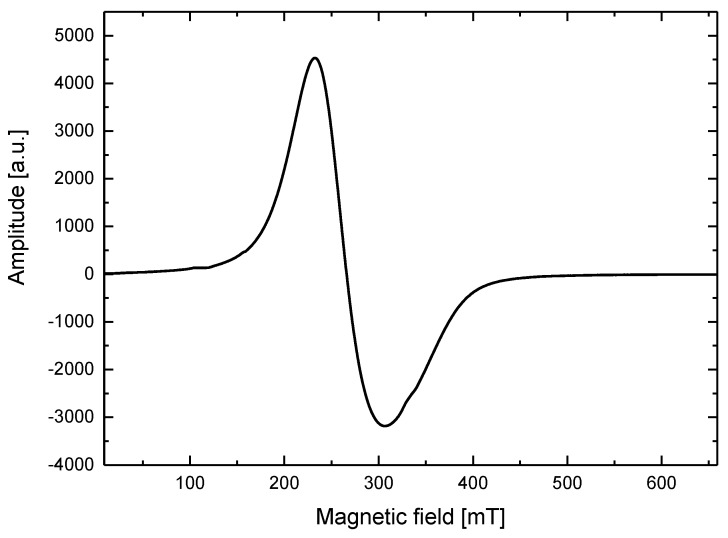
Electron spin resonance spectrum for the magnetic fluid.

**Figure 3:**
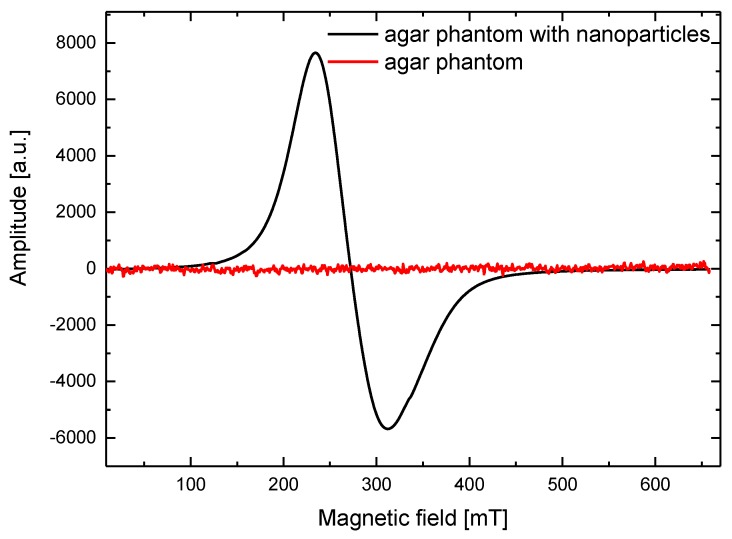
Electron spin resonance spectra for the pure agar phantom and agar phantom with nanoparticles.

The ESR measurements were performed using an X-band (9.4 GHz) Bruker ESR/ENDOR EMX-10 spectrometer (BRUKER, Billerica, MA, USA) with a 100 kHz magnetic-field modulation. The ESR spectra were acquired at room temperature for an amplitude modulation of 1 mT. The spectroscopic parameters, *g*-factor (spectroscopic splitting factor) and peak-to-peak line width (Δ*H*) with accuracies of ±0.005 and ±0.5 mT, respectively, were determined for each ESR spectrum. An ESR spectrum for the magnetic fluid is presented in Figure 2, while ESR spectra for the pure agar phantom and phantom with nanoparticles are presented in Figure 3. The spectroscopic parameters determined using the ESR spectra of the synthetized magnetic fluid, diluted magnetic fluid, and agar phantom with the same concentration of nanoparticles are presented in Table 1.

Figure 3 reveals that the pure agar phantom without magnetic material inside does not exhibit magnetic properties; therefore, no ESR spectrum was observed. The ESR spectra for both magnetic fluid (Figure 2) and magnetic nanoparticles in the phantom (Figure 3) are similar in shape; however, their spectroscopic parameters are different (Table 1). The highest *g*-factor value is observed for the original sample, while the lowest is observed for the nanoparticles diluted in water. For the line width (Δ*H*), the behavior is opposite, i.e., the line is the widest for the magnetic phantom with nanoparticles, while the narrowest line is observed for the original sample. In a concentrated sample (magnetic fluid), there are more nanoparticles in a given volume; therefore, they can aggregate and create larger structures. In such situation, the exchange interactions between nanoparticles increase, manifested by the narrowing of the ESR signal. Once the dilution nanoparticles are separated, the exchange interactions decrease, the line width increases, and the *g*-factor decreases. This is observed in the phantom where the magnetic nanoparticles are separated and their interactions are limited by the agar gel. It can be assumed that the prepared phantoms have homogeneous structure and distribution of nanoparticles. The spectroscopic parameters (Table 1) reveal that the employed magnetic fluid does not contain only pure magnetite nanoparticles; it is likely that there is a mixture of different iron-oxide nanoparticles such as magnetite and maghemite nanoparticles [33]. The shape of the ESR line and its anisotropy are typical for nanoparticles in the size range of 5–15 nm, presented in Figure 1.

### 2.4. Measurement Setup

**Figure 4:**
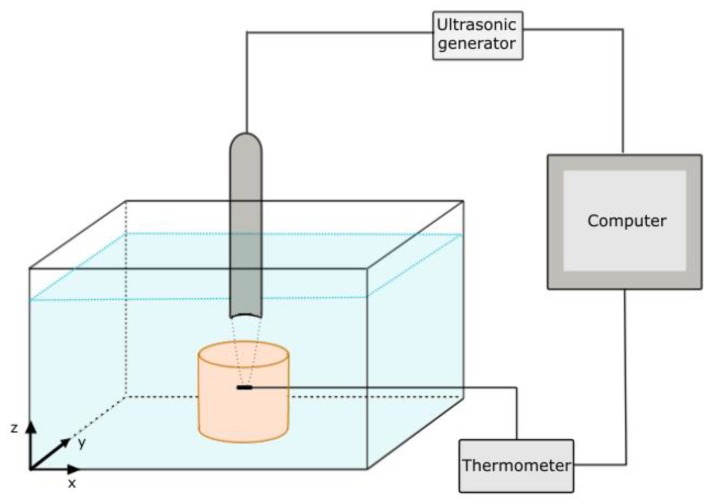
Schematic illustration of the focused ultrasound hyperthermia measurement setup.

In-vitro hyperthermia experiments were performed on the tissue-mimicking phantoms using a spherically focused single-element transducer fabricated by the Optel company. The measurement setup consisted of an ultrasound phantom placed in a plastic beaker filled with distilled and degassed water at room temperature. The ultrasound head immersed in the water was coupled to an ultrasound power generator, connected to a computer with a hyperthermia measurement software. The temperature in the phantom during the FUS hyperthermia was measured using a digital thermometer (Evolution FISO FPI-HR, FISO Technologies Inc., St-Jean-Baptiste, QC, Canada) with an optical fiber sensor (model FOT-L-SD, FISO Technologies Inc., St-Jean-Baptiste, QC, Canada). The optical fiber was centrally placed in the phantom at a height of approximately 0.5 cm below the phantom surface. The thermometer was connected to a computer to record the experimental data. The measurement setup is illustrated in Figure 4. The ultrasound transducer was driven in the continuous-wave mode with an operating frequency of 1 MHz. The acoustic power of the transducer was in the range of 2.7–10.3 W; the FUS hyperthermia results were obtained in this ultrasound power range. In order to position the thermometer as close as possible to the focus of the ultrasound beam, the phantom (containing the optical fiber) was mounted on a tri-axis positioning system (*x*, *y*, *z*). After locating the focus point, hyperthermia measurements were performed as a function of the acoustic power. In the experiments performed to evaluate the temperature as a function of the distance from the focus point, after 15 s of hyperthermia treatment, the transducer was manually moved away from the focus point by steps of 1 mm, first along the *x*-axis, and then along the *y*-axis. This enabled to record the temperature increase in the area near the focal zone.

## 3. Results

### 3.1. Heating in the Focal Area by FUS

The interaction of the ultrasound with the tissue depends on various parameters including the acoustic power (intensity), pressure amplitude, frequency, and sonication time. The effect of the magnetic nanoparticles on the rate of temperature increase in the focus point for different acoustic powers was investigated. The interaction between the ultrasonic waves and the solid particles of nanometer size suspended in the continuous media (gas, carrier liquid, gel matrix) leads to the additional attenuation of sound compared to that in the continuous phase. Three mechanisms are connected to this interaction: differences in compressibility, thermal properties, and density between dispersed particles and continuous phase. This additional attenuation strongly depends on the size of the particles and the frequency of the wave—increasing with both factors in the long-wavelength region, that is for kR≪1 (*k* being the wave number and *R*—the radius of the particles). The increase of the overall ultrasound attenuation, μ, results in tissue mimicking phantoms with nanoparticles suspended in gel-matrix results in more heat deposed into the phantom by ultrasound wave according to the well-known relation P=2μI, where P is the density of the heat power deposed in the phantom by the ultrasound wave of the intensity I.

**Figure 5:**
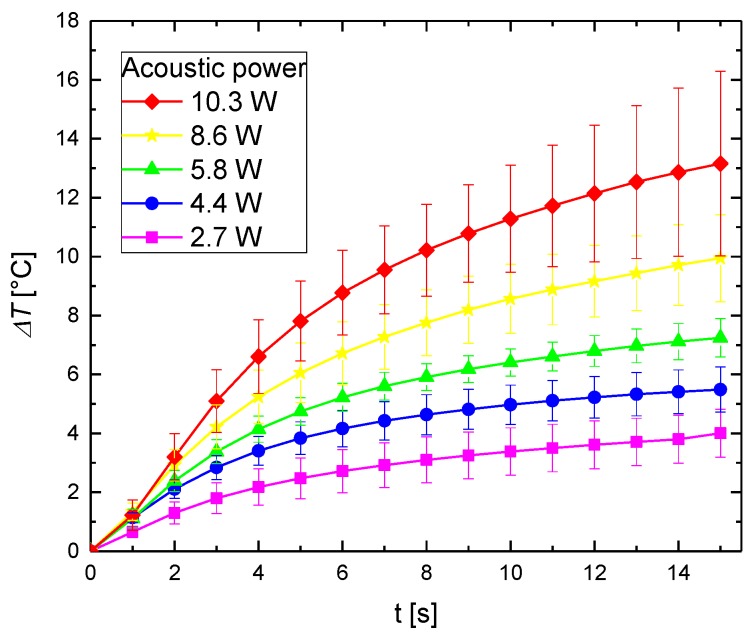
Experimental results of the temperature increase as a function of the time and acoustic power for 5% concentration of agar gel during the 1-MHz ultrasound sonication.

Measurements were repeated a minimum of 5 times and provided results represent the average value of temperature increase. The experimental results of the temperature variations as a function of the time for the pure agar phantom at different acoustic powers are presented in Figure 5. The increase in the phantom temperature is caused by the attenuation of the ultrasound wave. A part of the mechanical energy is lost owing to its conversion to heat. The temperature increases with the acoustic power. A higher temperature increase is caused by attenuation of a larger amount of ultrasound energy.

**Figure 6:**
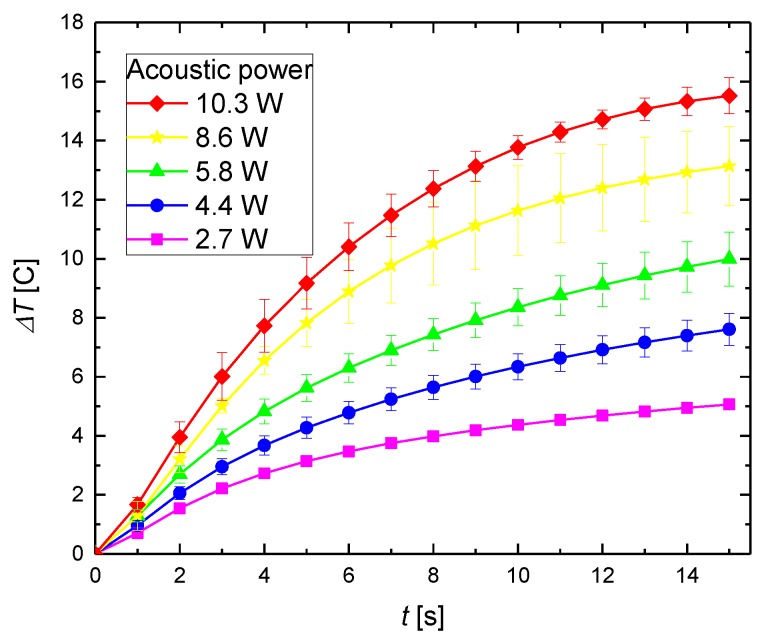
Experimental results of the temperature increase as a function of the time and acoustic power for 5% concentration of agar gel with magnetic nanoparticles (*ϕ_M_* = 0.35% (*w*/*w*)) during the 1-MHz ultrasound sonication.

It is worth to perform thermal studies on the phantoms doped with sonosensitizing nanomaterial. The addition of nanoparticles changes the attenuation coefficient of the medium, and consequently the rate of heating [33]. The temperature variations with time for the phantom with magnetic nanoparticles are presented in Figure 6.

For the agar phantom with magnetic material, the temperature also increases with the acoustic power. However, the agar phantom doped with sonosensitizers exhibits a higher temperature increase, compared with the pure agar phantom for the same sonication time. The higher temperature increase emerges owing to the additional attenuation of the scattering magnetic material.

**Figure 7:**
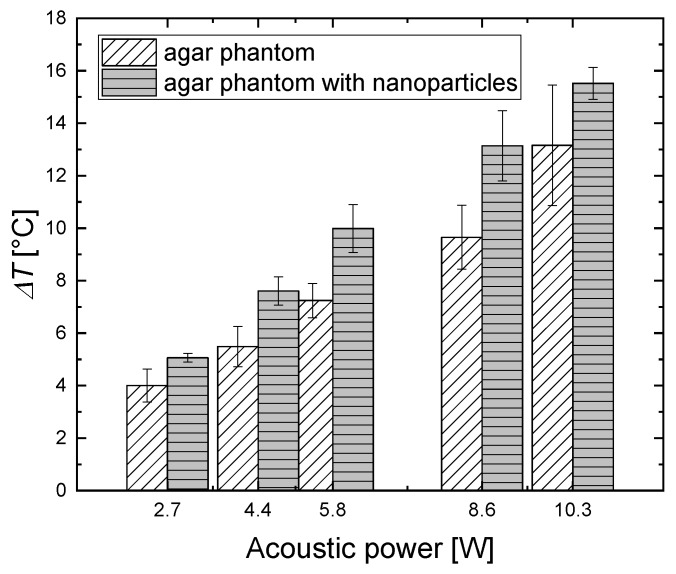
Temperature increase for a 5% concentration of pure agar gel and agar gel with magnetic nanoparticles (*ϕ*_M_ = 0.35% (*w*/*w*)) for an ultrasound frequency of 1 MHz for different acoustic intensities.

Figure 7 shows the ultrasound-induced temperature variations with time for the pure agar phantom and agar phantom with magnetic material for different acoustic powers. The temperature increase in phantoms with and without magnetic nanoparticles were each compared using a paired two-tailed *t*-test. A difference of the means with *p* < 0.05 was taken to be significant. For example, the mean temperature differences between the phantoms with and without magnetic nanoparticles were significant (*p <* 0.02) for 10.3 W of acoustic power and highly significant for 5.8 W and 2.7 W (*p* < 0.000002 and *p* < 0.0000005, respectively). Thus, it can be concluded that the end-of-sonication temperature for a magnetic-particle concentration of *ϕ_M_* = 0.35% is higher than that for the phantom containing only agar, for all acoustic powers.

This clearly demonstrates that magnetic nanoparticles are a good sonosensitizing material not only for planar ultrasound hyperthermia [33] but also for FUS therapy.

### 3.2. Heating by the FUS in an Area Distant from the Focal Point

**Figure 8:**
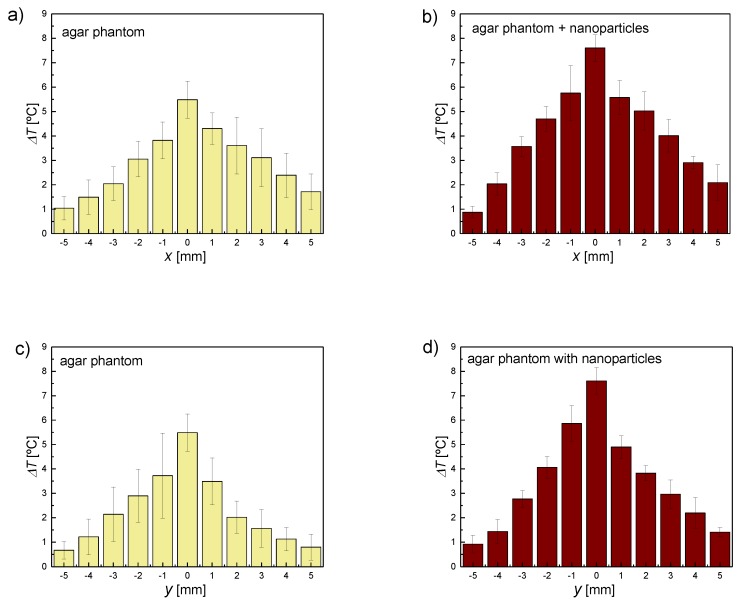
Experimental temperature increase after 15 s of sonication as a function of the distance from the center of the focus point along the (**a**) and (**b**) *x*-axis; (**c**) and (**d**) *y*-axis.

In thermal therapies, it is crucial to increase the temperature in a specific area without affecting the surrounding healthy tissues. Therefore, in HIFU treatments, the shape and size of the focal area, in which the temperature is increased, are essential. In order to investigate the heat propagation in the ultrasound phantom in the focus area and effectiveness of the ultrasounds in the center of the focus region, additional experiments were performed. After positioning the thermometer as close as possible to the center of the ultrasound beam, experiments were performed at an acoustic power of 4.4 W to evaluate the temperature change as a function of the distance from the focus center. Figure 8a,b present the results of the FUS hyperthermia as a function of the *x*-distance, while Figure 8c,d present the results of the FUS hyperthermia as a function of the y-distance from the center of the focus point for the pure agar phantom and that doped with sonosensitizers, respectively. Differences among means are statistically significant (*p* < 0.05) by one-way ANOVA.

The experimental results presented above clearly show that the temperature increase induced by the FUS decreases with the increase of the distance from the center of the focus point. Moreover, the temperature increase obtained for the phantom with magnetic material was higher than that for the pure agar phantom. The most noticeable temperature increase was obtained in the center of the focus area.

### 3.3. SAR

One of the most common methods to characterize the power deposition in the tissue during the thermal treatment is to evaluate the SAR. The SAR value depends on the efficiency of the heat source and the ability of the heated medium to absorb thermal energy. It means that in case of ultrasound heating the SAR value depends on the ultrasound intensity in the focal zone and the coefficient of ultrasound absorption of the heated region. The accurate determination of the SAR during the FUS heating provides a physical basis to compare the temperatures obtained in the phantoms with and without nanoparticles. According to the usual definition, the SAR describes the rate of energy absorption by a tissue, calculated as [40]:

(2) $$\mathrm{SAR}=c_{p}{\left(\frac{dT}{dt}\right)}_{t=0}$$

where *c_p_* is the specific heat of the ultrasound phantom. In practice, obtaining SAR experimentally involves measuring temperature increase following a step-in heating, fitting the resulting data to a linear function in time, and then determining its slope at time zero. SAR may only be accurately evaluated from initial slope of the temperature rise. This method does not take into account heat conduction losses. This assumption may be valid if heat conduction losses are small compared to the applied power. However, in FUS treatments with their small focal zones, it could be expected that heat conduction might become important at very short times after heating is initiated, raising doubts about the accuracy and practicality of the linear fit method.

Recently, a new analytical solution of the heat transfer equation for an improved SAR estimation has been proposed by Dillon et al. [40]. Their analytical model assumes that the radial distribution of the ultrasound power deposition pattern has a Gaussian shape:

(3) $$Q=2\alpha I_{0}\;\exp\left(\frac{-r^{2}}{\beta }\right)$$

where the parameter β is a measure of the spread of the focal region. If the ratio of the axial to lateral beam widths is larger than 2, one may assume that the spatial temperature gradient is dominated by the radial conduction, and that the heat conduction in the axial direction can be neglected. According to the Dillon’s analytical model, if off-axis experimental temperatures are measured as a function of time, they could be used to directly estimate the SAR for the entire heating plane. The estimation of the SAR values from these experimental data requires the off-axis analytical step-heating solution of the heat transfer equation, i.e., for r≠0. It has the following form:

(4) $$T\left(r,t\right)=C\left(\frac{\beta }{4\kappa }\right)\left[\mathrm{Ei}\left(\frac{-r^{2}}{\beta }\right)-\mathrm{Ei}\left(\frac{-r^{2}}{\beta (1+4\kappa t/\beta }\right)\right]$$

where $C=2\alpha I_{0}/\rho c_{p}$ and Ei represents the exponential integral. The acoustic pressure absorption coefficient of the tissue is represented by *α* in the term *C*; *I*_0_ is the initial ultrasound intensity along the beam axis, *ρ* is the tissue density, and *c_p_* is the specific heat of the tissue [41].

By a simultaneous fitting of all of the temperature–time data measured off-axis using Equation (4), one can obtain single estimates for *C*, thermal diffusivity *κ*, and Gaussian variance *β*. The on- and off-axis SAR values can then be calculated using:

(5) $$\mathrm{SAR}\left(r\right)=c_{p}C\cdot \exp\left(\frac{r^{2}}{\beta }\right)$$

According to the data sheet of the transducer the lateral full width at half maximum (FWHM) was assumed to be 0.9 mm which corresponds to the parameter $\beta =2.9\times 10^{-7}\;m$. In general β may vary in case of inhomogeneous phantoms but because of the small concentration of sonosensitizing nanoparticles (*ϕ_M_* = 0.35%), it was assumed β to be the same for pure agar sample and agar with magnetic nanoparticles. This left only two parameters C and κ to be fitted. From the least-squares fit of experimental temperature-time data to the Equation (4) the thermal diffusivities of the pure agar phantom and agar phantom with magnetic nanoparticles were estimated at $\left(1.5\pm 0.1\right)\times 10^{-7}{\;m}^{2}\;s^{-1}$ and $\left(2.8\pm 0.2\right)\times 10^{-7}{\;m}^{2}\;s^{-1}$, respectively. These values are in good agreement with our own assessment [33].

**Figure 9:**
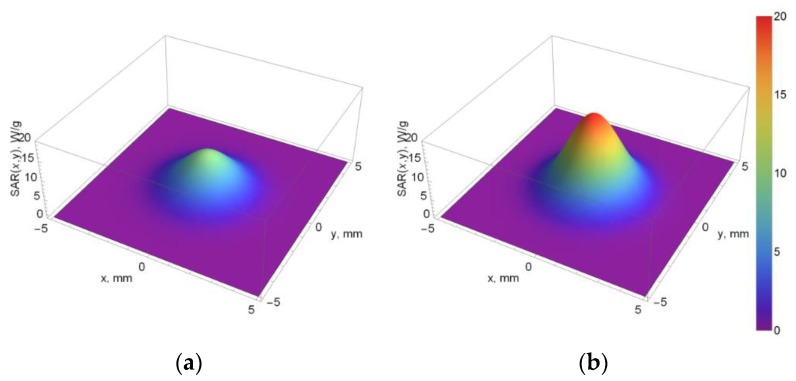
SAR distribution in focal plane calculated from the fitting of the off-axis temperature–time data with Equation (4): (**a**) Pure agar sample; (**b**) Agar phantom with magnetic nanoparticles with a concentration of *ϕ_M_* = 0.35%.

Using the values of C and κ obtained from the best-fit procedure for the agar sample and agar with magnetic nanoparticles, respectively. The addition of the magnetic material increases estimated SAR value in the center of the focal zone (red area in Figure 9b) compared to that in pure agar sample. Outside the center of the focal zone, the estimated SAR value rapidly goes away. Therefore, it can be inferred that a tissue outside the focal zone will not be overheated.

Table 2 presents the SAR values estimated for the center of the focal zone and at various radial distances from it. For the pure agar phantom and agar phantom with magnetic nanoparticles, the highest SAR values were obtained in the center of the ultrasonic beam. With the increase of the distance from the center of the ultrasound focus, the SAR decreased.

The global SAR values describing the overall power deposition in the tissue during the thermal treatments for the pure agar phantom and agar phantom with sonosensitizers were calculated to be 9983 W/kg and 19765 W/kg, respectively. These results show that the addition of nanoparticles almost doubles (increase of 98%) the global SAR, compared to that for the pure phantom. The increase in final temperatures (after 15 s of heating) between both samples is less than increase in the SAR values (38% vs. 98%). The reason for that is the thermal diffusivity which is about two times higher for the agar phantom with nanoparticles compared to that for the pure agar sample. This results in faster heat dissipation in case of phantom with magnetic nanoparticles.

### 3.4. Ultrasound Imaging

**Figure 10:**
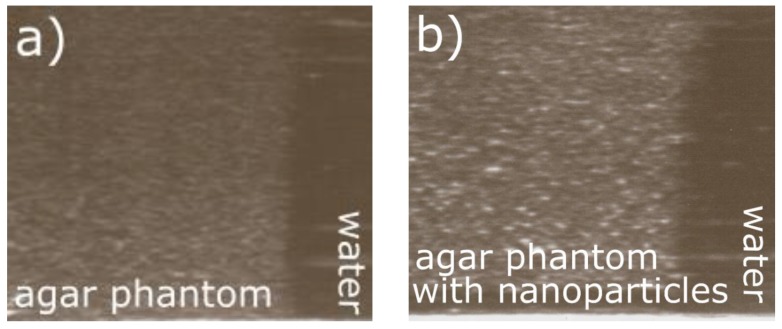
Ultrasound images of the (**a**) pure agar phantom; (**b**) Agar phantom with magnetic nanoparticles.

Ultrasounds can be used not only for therapy but also for imaging and monitoring of the therapeutic process and efficiency. Ultrasonography is one of the most common imaging methods in clinical applications owing to its high sensitivity, broad accessibility, portability, and low cost. Commonly used ultrasound contrast agents, microscale bubbles, are too large to extravasate from the vascular lumen. The smaller-size nanoscale agent is advantageous in this regard and mediates their accumulation at target sites [42]. The studied agar gel phantoms without any added scattering material and agar gel phantoms with magnetic nanoparticles were scanned with a diagnostic ultrasound imaging system (Sonoline Prima, SIEMENS, Munich, Germany). The pure agar phantom was homogeneously hypoechoic owing to the lack of scattering (Figure 10a). The effect of the scattering-material addition was manifested in the changes of the ultrasound wave parameters. The values of the acoustic impedance *Z =* *c·ρ* (*c*: ultrasound velocity, *ρ*: density) also increased after the addition of nanoparticles [43]. The phantoms with magnetic nanoparticles exhibited an increased echogenicity owing to the significant number of scatters (Figure 10b) [44]. However small amount of magnetic nanoparticles alone is a weak contrast agent to ultrasound imaging. Nanoparticles are too small to backscatter ultrasound at a detectable level [45]. However, it is possible to use magnetic properties of nanoparticles for molecular imaging by using the motion of these particles when subjected to gradient magnetic field. Magnetic material placed in a nonuniform magnetic field, will rotate until its magnetic moment is parallel to the field and translate toward the region with greater magnetic field strength. The nanoparticles will be set in motion, and this movement (i.e., magnetomotive movement) can be detected with ultrasound. Particle motion sensitizes ultrasound imaging systems [45,46,47]. It can be stated that the magnetic nanoparticles, which increase the therapeutic efficacy of the FUS hyperthermia, can also influence the ultrasound contrast imaging (ultrasonography), and can act as theranostic nanoparticles.

## 4. Conclusions

The experimental results and numerical analysis showed that magnetic nanoparticles could be employed as sonosensitizing materials in therapeutic FUS hyperthermia. Their presence in tissue-mimicking phantoms changed the rate of heating. The addition of the nanoparticles increased the global SAR almost two times compared to that for the pure phantom. The temperature increase induced by the FUS and SAR evaluated from the experimental data decreased with the increase of the distance from the center of the ultrasound focal zone. Therefore, the use of sonosensitizers in FUS hyperthermia is advantageous as the temperature in the focal point sufficiently increases, while the surrounding area is not overheated. In addition, the desired temperature increase in the focus can be obtained in a shorter time, compared to pure FUS hyperthermia. Ultrasound imaging studies showed that magnetic nanoparticles, in addition to their ability to increase the therapeutic efficacy of FUS hyperthermia, can improve the contrast in ultrasonography. Therefore, magnetic nanoparticles are very good candidates for ultrasound theranostic therapy.

## Figures and Tables

**Table 1:** Spectroscopic parameters determined from the ESR spectra of the studied samples.

Sample	*g*-Factor ± 0.005	Δ*H* ± 0.5 (mT)
Magnetic fluid	2.521	75.5
Magnetic fluid diluted in water (0.35% (*w*/*w*))	2.437	79.1
Agar phantom	–	–
Agar phantom with nanoparticles (0.35% (*w*/*w*))	2.461	79.6

**Table 2:** SAR values of the FUS hyperthermia along the radial direction from the center of the focus zone.

Distance from Focus (mm)	SAR for Pure Phantom (W/kg)	SAR for Phantom with Sonosensitizers (W/kg)
5	2	5
4	48	95
3	497	984
2	2631	5210
1	7153	14,162
0	9983	19,765
